# MetaboNetworks, an interactive Matlab-based toolbox for creating, customizing and exploring sub-networks from KEGG

**DOI:** 10.1093/bioinformatics/btt612

**Published:** 2013-10-30

**Authors:** Joram M. Posma, Steven L. Robinette, Elaine Holmes, Jeremy K. Nicholson

**Affiliations:** Computational and Systems Medicine, Department of Surgery and Cancer, Faculty of Medicine, Imperial College London, SW7 2AZ London, UK

## Abstract

**Summary:** MetaboNetworks is a tool to create custom sub-networks in Matlab using main reaction pairs as defined by the Kyoto Encyclopaedia of Genes and Genomes and can be used to explore transgenomic interactions, for example mammalian and bacterial associations. It calculates the shortest path between a set of metabolites (e.g. biomarkers from a metabonomic study) and plots the connectivity between metabolites as links in a network graph. The resulting graph can be edited and explored interactively. Furthermore, nodes and edges in the graph are linked to the Kyoto Encyclopaedia of Genes and Genomes compound and reaction pair web pages.

**Availability and implementation:** MetaboNetworks is available from http://www.mathworks.com/matlabcentral/fileexchange/42684.

**Contact:** jmp111@ic.ac.uk or j.nicholson@imperial.ac.uk

**Supplementary information:** Supplementary data are available at *Bioinformatics* online.

## 1 INTRODUCTION

Investigating the dynamic metabolic responses in living systems because of external perturbations gives complementary information to genomic and/or proteomic approaches. This field is known as metabonomics (Nicholson *et al.*, 1999) and provides a ‘top-down’ view of the living system to show global changes instead of cell-specific interactions between genes, proteins and/or metabolites. Metabolic pathway information can aid the interpretation of biological changes indicated by significant differences in metabolite concentration identified in metabonomic studies. The effective transformation of metabolic spectroscopy data to biological knowledge presents a significant bioinformatics challenge. In particular, the increased interest in clinical applications of metabolic phenotyping makes practical data visualization in a biological/medical framework of great importance.

The Kyoto Encyclopaedia of Genes and Genomes (KEGG) (Kanehisa and Goto, 2000) is an online resource where the interaction information of genes, proteins and metabolites is integrated and it can be used to investigate molecular networks in specific organisms or for all (Kanehisa *et al.*, 2012). KEGG provides many static pathways and metabolic reaction networks as well as a global metabolic map. The standalone application NeAT (Faust *et al.*, 2010) can be used to draw custom metabolic reaction sub-networks for a specific organism. However, it is useful to have the ability to edit graphs, as can be done for pathways e.g. using KEGGParser (Arakelyan and Nersisyan, 2013) in Matlab. MetaboNetworks aims to fill this gap for metabolic reaction networks and combine the ability to draw custom maps and being able to edit them.

Rarely do metabolic networks and programs consider that the typical mammal is not a single organism, but a system comprising of a combination of mammalian, bacterial and potential parasitic organisms. Specifically, there is symbiosis between a host organism and its gut microbiota, and that gut microbes have functions and enzymes that are not found in mammalian organisms (Gill *et al.*, 2006; Nicholson *et al.*, 2012). Therefore, it is important to be able to combine metabolic reaction networks for different organisms. MetaboNetworks has the option to include data from multiple organisms and investigates if reactions can occur in any of the selected organisms. In the present study, we have illustrated the new approach using an NMR-based toxicological biomarker input dataset. This is an area of particular importance as metabolic phenotyping has shown to be particularly useful for studying toxicological processes as models for human disease (Nicholson and Wilson, 2003; Nicholson *et al.*, 2002).

MetaboNetworks only requires the basic version of Matlab (The Mathworks, Natick, MA, USA) and does not require any additional toolbox. Although the MatlabBGL toolbox (Gleich, 2006) has a number of graph layout algorithms in mex/c++ code that can be used to speed up the calculation of large graphs in MetaboNetworks, all MetaboNetworks’ functionalities are implemented using a simple graphical user interface and Matlab code.

## 2 METHODS AND FEATURES

First, the appropriate data from KEGG has to be imported to Matlab. In MetaboNetworks this is done using a function that uses the KEGG REST-API to calculate a metabolite adjacency matrix that can later be used to draw the graphs. The user can select one or multiple organisms for which complete genomes are available in KEGG; for these organisms a list of enzymes (with E.C. numbers) that are associated with a gene from any of the organisms is determined. Using this enzyme list, all reactions are queried and enzymes involved in the reactions are matched against the enzyme list. Only reactions that require an enzyme from the list or that are listed as ‘non-enzymatic’ or ‘spontaneous’ are used to find their main reaction pairs. The compounds from these reaction pairs are considered adjacent. Each row/column in the adjacency matrix indicates a specific compound (with a KEGG compound ID) and a list of all names for these compounds are found from the KEGG compound database. A reaction database has previously been collected using a similar approach (Ma and Zeng, 2003) to MetaboNetworks, however, that database includes reactions from all species, whereas MetaboNetworks focusses on organisms of interest as not all reactions can occur in all organisms.

Second, when the data collection is complete, MetaboNetworks can be used to create and explore custom networks. A list of metabolites, e.g. biomarkers arising from a metabonomic experiment, can be passed to MetaboNetworks and it searches for the shortest path between each of these metabolites using the breadth-first search algorithm. All compounds that are a part of a shortest path between any of the metabolites are included in the network. By default, MetaboNetworks plots the network as a circular graph. Other graph layouts include a spring-embedded layout, high-dimensional embedding and two types of uniform edge-length layouts, the last aim to place nodes with as little overlap as possible. If the Matlab statistical toolbox is installed, multidimensional scaling can also be used.

**Fig. 1.**
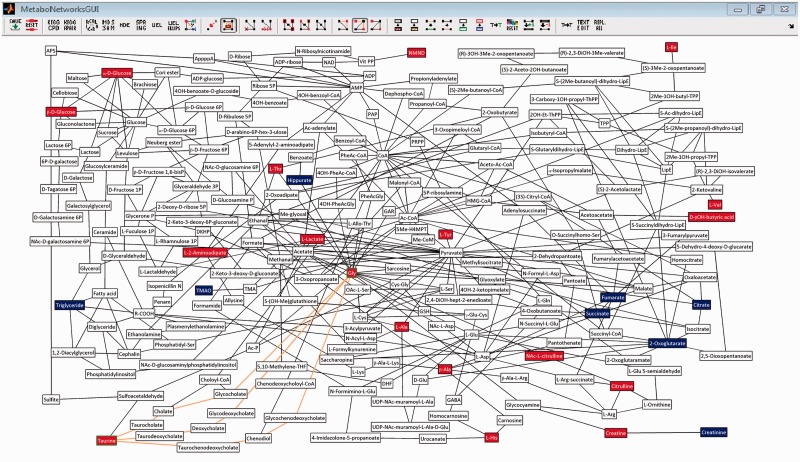
Shows the graphical user-interface of MetaboNetworks with a custom network drawn for significant metabolites from a hydrazine toxicity study in rats (Nicholls *et al.*, 2001). Metabolites higher in hydrazine-dosed rats compared with controls are shown in red, and metabolites lower in hydrazine-dosed rats are shown in blue. The white nodes are part of shortest paths between the coloured nodes. The edges shown in orange are part of the shortest path (four reactions) between taurine and glycine. Aside from the rat, all bacteroidetes and firmicutes species were included in the database

Last, when the initial network layout is satisfactory the graph layout can be manually adjusted. Supported adjustments include node position, node/edge removal, highlighting nodes (see green edges of nodes in Fig. 1), and shortest paths (orange edges in Fig. 1), node text and nodes/edge/text properties (font, width, size, etc.). If additional data is supplied, the association of the metabolites with a response variable can be shown as node colour (see Fig. 1). Furthermore, the network can be exported as a tif, png, pdf, eps or other image formats, the network can always be reset to the original graph (all changes are lost). Another option is to click on a node to open a web browser showing the compound entry in KEGG or show reactions pairs in KEGG of selected nodes. The Supplementary Information includes a full walkthrough of the software and all the capabilities.

## 3 DISCUSSION

MetaboNetworks can be used to investigate and modify complex metabolic reaction networks. It improves the visual interpretation of metabonomic experiments with coverage across disparate metabolic pathways. The network should ideally be viewed as a connected network of probabilistic events (reactions) (Wilson and Nicholson, 2003) that lie on the shortest path between two metabolites. The database can be tailored to the study by including specific organisms, as we have done for the hydrazine toxicity network by including all bacteroidetes and firmicutes species, as it has been shown that germ-free rats react differently to hydrazine (Swann *et al.*, 2009). MetaboNetworks can possibly also be used for identifying unknown metabolites by generating a network of metabolites correlated to the unknown metabolite. The unknown can be part of the shortest path between the known metabolites as the correlation can be due to a similarity in structure.

Note that use of the KEGG-API is free for academic use; however, non-academic users should ask for permission (see http://www.pathway.jp/).

*Funding*: Medical Research Council - Public Health England (MRC-PHE) Centre for Environment & Health PhD-studentship (to J.M.P.). We thank the Medical Research Council - National Institute for Health Research (MRC-NIHR) National Phenome Centre for funding this and related work.

*Conflict of interest*: none declared.

## Supplementary Material

Supplementary Data
