# Supplementary material for: MetaboNetworks, an interactive Matlab-based toolbox for creating, customizing and exploring sub-networks from KEGG
Source: Bioinformatics. 2013 Oct 30;30(6):893–5. doi: 10.1093/bioinformatics/btt612 (PMC3957072; doi:10.1093/bioinformatics/btt612)
Supplement: Supplementary Data [file supp_30_6_893__index.html]

MetaboNetworks, an interactive Matlab-based toolbox for creating, customizing and exploring sub-networks from KEGG — MetaboNetworks, an interactive Matlab-based toolbox for creating, customizing and exploring sub-networks from KEGG — MetaboNetworks, an interactive Matlab-based toolbox for creating, customizing and exploring sub-networks from KEGG — Supplementary Data 

# MetaboNetworks, an interactive Matlab-based toolbox for creating, customizing and exploring sub-networks from KEGG

## Supplementary Data

files

**Files in this Data Supplement:**

- Supplementary Data - doc file
