# Supplementary material for: MetaboNetworks, an interactive Matlab-based toolbox for creating, customizing and exploring sub-networks from KEGG
Source: Bioinformatics. 2013 Oct 30;30(6):893–5. doi: 10.1093/bioinformatics/btt612 (PMC3957072; doi:10.1093/bioinformatics/btt612)

MetaboNetworks, an interactive Matlab-based toolbox for creating, customizing and exploring sub-networks from KEGG

Joram M. Posma^1, *^, Steven L. Robinette^1^, Elaine Holmes^1^ and Jeremy K. Nicholson^1, *^

^1^ Computational and Systems Medicine, Department of Surgery and Cancer, Faculty of Medicine, Imperial College London, SW7 2AZ, London, United Kingdom

**Supplementary material**

First, we show a high-resolution version of the network shown the main paper. With the toolbar buttons explained as they appear from left to right. Second, we describe the functionalities of MetaboNetworks in more detail and provide a walkthrough guide on how to go from database creation to customizing and exploring the metabolic reaction network.


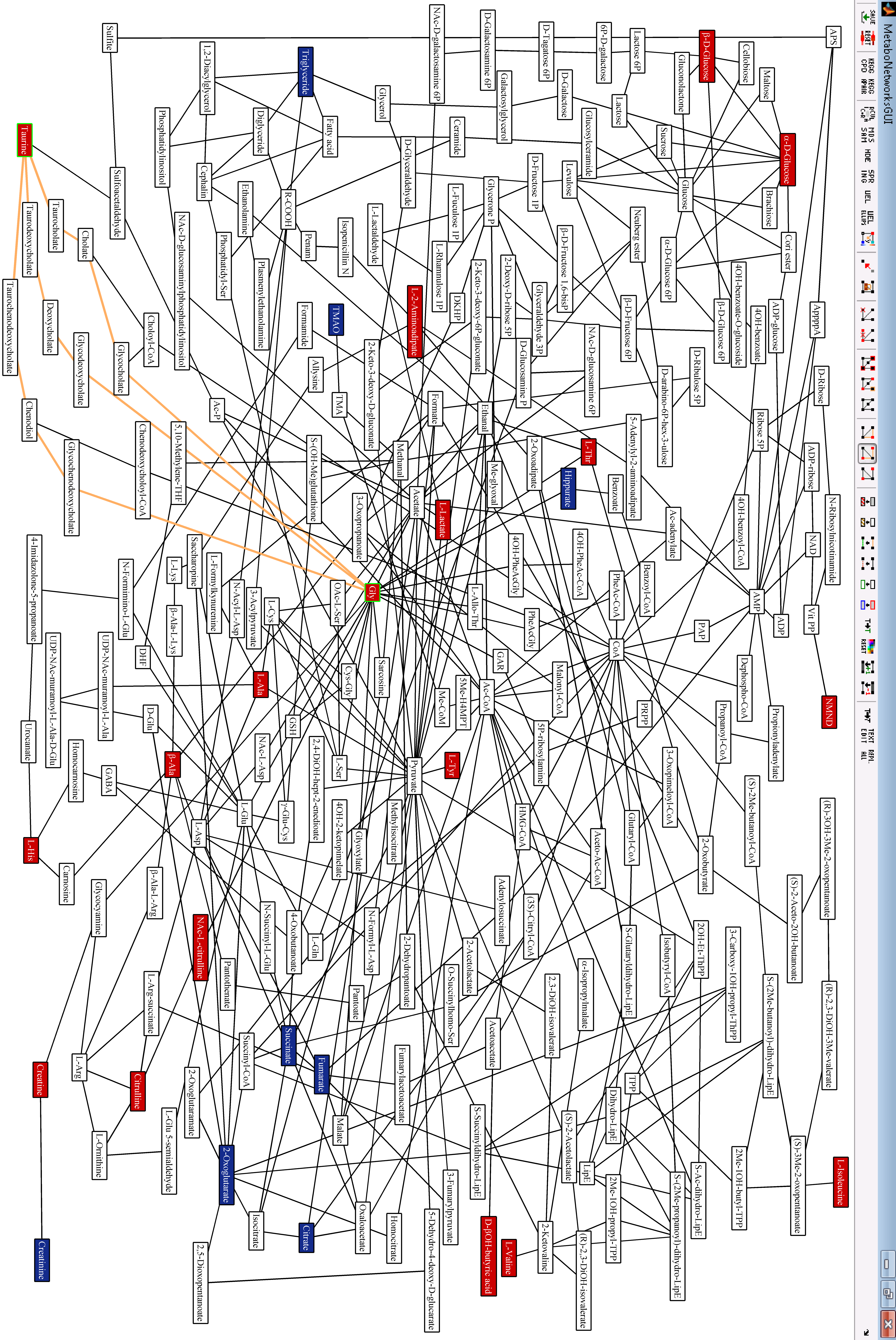


Toolbar buttons, from left to right

Save – Opens a save dialog screen where the user can choose the filename, location and image type. All default image formats in Matlab can be chosen: bmp, eps, emf, jpg, pcx, pbm, pdf, pgm, png, ppm and tif. As well as the Matlab image format .fig. This will automatically save the database in the same location with the same name. The .fig can be opened, the database selected and the user can continue editing and exploring the network from where it was saved.

Reset – This will reset the network to the original network; all node and edge deletions, node positions, and other changes are lost.

KEGG compound – This lets the user pick a metabolite and will open a web browser showing the KEGG compound page for the metabolites.

KEGG reaction pair – This will let the user pick two metabolites and depending on whether there is a link in the network, a web browser will be opened showing the reaction pair web page for the selected metabolites. If multiple metabolites are selected prior to pressing the button, MetaboNetworks will evaluate which are directly linked and open all reaction pairs in one web page.

Circular layout – This is the default layout, nodes are placed on a circle starting with the supplied metabolites. The metabolite name is displayed on the outside of the circle.

Multi-dimensional scaling (MDS) – This layout can only be chosen if the Matlab statistics toolbox has been installed, if not this is disabled. It uses multi-dimensional scaling to calculate node positions using the distance matrix as similarity matrix.

High-dimensional embedding (HDE) – This calculates node positions based on the first two scores of principal component analysis using the adjacency matrix as input.

Spring-electrical embedding (SEL) – This uses the adjacency matrix to calculate the node positions aiming to minimize the spring-electrical forces. This is an iterative process and can take a long time when the network is large. The user can exit the optimization by pressing the ok button. The algorithm will finish the current iteration and show the node positions. Ordinary spring-type algorithms will not be able to cope with networks that are not fully connected. SEL places nodes without connections on the outside of the network.

Uniform edge length (UEL) – This uses the distance matrix to calculate node positions, it starts with the node with the highest degree which is placed in the centre. In each following step the metabolite with the highest degree is placed at a position which is as close to the actually distance as possible.

Uniform edge length ellipse – This algorithm uses the same procedure as the previous algorithm, except it takes into account the length of the text of the node. It aims to place nodes as far from each other as possible, while preserving the distances, to end up with as little overlap of node labels as possible. Overlap of node labels can become problematic for very large networks.

Colour nodes based on association – Optionally a vector the same size as the metabolite list can be supplied to MetaboNetworks. Each selected metabolite has a value, e.g. the correlation with the response variable of the study, the regression coefficient or other. This will change the node colours of selected metabolites based on the value on a continuous scale colours ranging from dark blue to white to dark red. If no vector is supplied, this option is disabled.

Move nodes – When enabled, the user can move nodes by holding the (left) mouse button down and releasing it where the node should be moved to. The node that was closest to the position of the mouse when the button was pressed is moved to the new location. The user can continue to move nodes as long as a movement or click is detected every 30 seconds. If there are 30 seconds of inactivity the function is disabled and needs to be enabled again. When the move node function is timed out, the node positions are saved and locked (see next button for description).

Lock node positions – This button locks the node positions until an action is performed which resets or changes node positions. The move node button automatically calls this function when timed out. This ensures the positions are fixed and are not changed when other functions are called which may call graph layout algorithms.

Delete (selected) node(s) – This will allow the user to select a node to delete from the network, this will immediately recalculate the node positions using the same layout algorithm as was used to calculate the previous network. To delete multiple nodes at once, the user must select nodes first (see select nodes button). This will result in new node positions, the previous ones (locked or not) will not be saved.

Delete edges between (selected) nodes – This will ask the user to choose 2 nodes in the network, if there is an edge between them this will be removed and a new network will be calculated. If multiple nodes are selected, this will remove all edges between them.

Select nodes – This will let the user select nodes in the network, at each mouse click the node closest to the position of the mouse will be added to the selection list. As with the move node button, there is a time-out of 30 seconds.

Deselect nodes – This will let the users deselect nodes. The selected node closest to the position of the mouse click will be de-selected.

Deselect all nodes – This will deselect all nodes at once.

Highlight degree of (selected) node(s) – The user is asked to select a node first after which all edges from this node are highlighted. If there are nodes in the network selected, this will automatically highlight all their edges.

Show shortest path between (selected) nodes – This will highlight the shortest path between selected nodes. If no nodes are selected, the user is asked to select two nodes. If 2 or more nodes are already selected, this will immediately show the shortest paths between them.

Un-do edge colouring – This will un-highlight the edges, either from the degree or shortest paths buttons.

Change node colours of supplied metabolites – By default the biomarker metabolites are coloured grey to distinguish them from the metabolites part of the shortest paths. Clicking this button will open a dialog box in which the user can choose another colour.

Change node colours of other metabolites – By default metabolites part of the shortest paths have a white background to distinguish them from the biomarker metabolites. Clicking this button will open a dialog box in which the user can choose another colour.

Change edge colour – By default the edges are shown in black. Clicking this button will open a dialog box in which the user can choose another colour for the edges.

Change edge colour of highlighted edges – In order to distinguish highlighted edges from non-highlighted edges, the default colour for highlighted edges is orange. Clicking this button will open a dialog box in which the user can choose another colour for showing the highlighted edges.

Change node box colour – By default the box colour of all nodes is black. Clicking this button will open a dialog box in which the user can choose another colour for the outer boxes of nodes.

Change node box colour of selected nodes – By default the box colour of selected nodes is red to distinguish between selected and un-selected nodes. Clicking this button will open a dialog box in which the user can choose another colour for the outer boxes of selected nodes.

Change colour of text – By default the text colour is black. Clicking this button will open a dialog box in which the user can choose another colour for the text on the nodes.

Reset colours – This resets all colours back to the defaults.

Increase line width – Increases the line widths of edges and node boxes by 1 point (default is 1, maximum is 5).

Decrease line width – Decreases the line widths of edges and node boxes by 1 point (minimum is 1).

Change font – This opens a dialog box in which the user can change the font type, size and emphasis (bold, italic).

Edit text of a node – This will ask the user to select a node first, after a node has been selected the text can be edited. This can be used to manually shorten names to create more space in the graph or to re-name metabolites.

Replace text in all nodes – This will allow the user to make changes to names of all nodes with a search string simultaneously, e.g. to change hydroxy to OH. Optionally, this process can be performed for a case-sensitive case by quoting the first string (“hydroxy”), this will not change ‘Hydroxy’ to OH, but only the lower case string.

Walkthrough guide

First the MetaboGetworks function has to be called from Matlab to start database collection:

>> MetaboGetworks

The software will now query KEGG for the names and classes of all complete genomes. Once it has finished a GUI pops up (OrganismFindGUI).


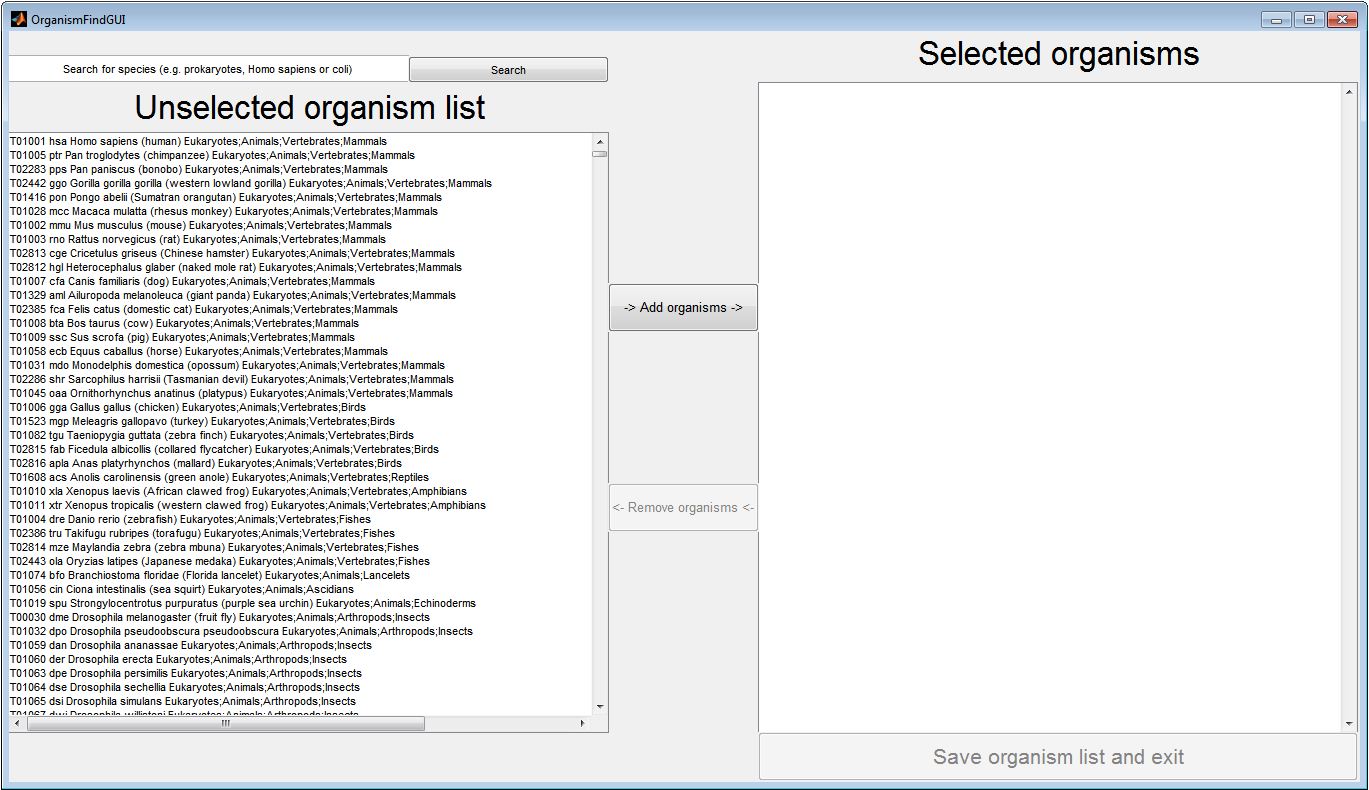


Here, the user can search for species to include. In the example below we searched for all firmicutes species and selected and added all lactococcus species from this list.


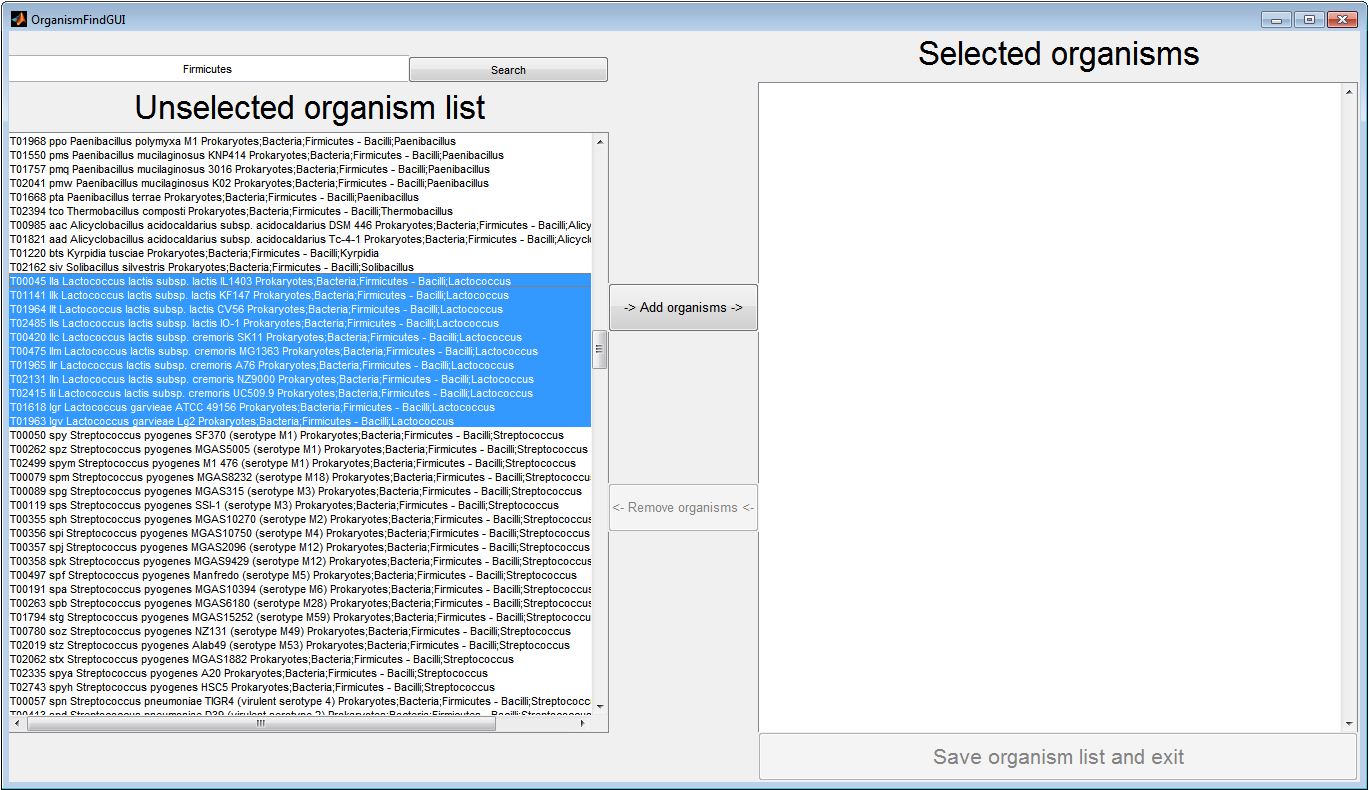


The user can always remove species from the selection list and to retrieve a list with all genomes the user can search for e.g. the letter T. Once the user is happy with the selection, we save the list of organisms and the user is queried to choose a name and location to save the database to. Once this has been chosen the software checks if the correct file format (.mat) has not been changed. If not, the software exits immediately, if everything is correct the software starts the process of collecting the database.

First, the software finds all enzymes (with E.C. numbers) associated with genes from the species by checking all associated pathways. A status bar pops up to show the progress.


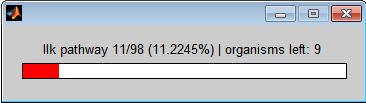


Second, the software loops through all reaction mains and investigates if the reaction pair is of type ‘main’, and if an enzyme from the list is involved or if the reaction is spontaneous/non-enzymatic. If the reaction pair satisfies these conditions the metabolites are considered adjacent.


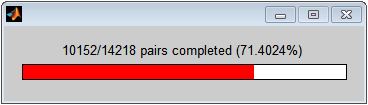


Last, the software loops through all compounds in KEGG and finds all their names in KEGG. This list is used by the MetaboNetworks sub-functions to search for metabolites later.


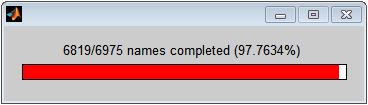


At this point the database is saved at the previously specified location with the chosen name. This process can take between 1 hour and 2 days depending on the number of species chosen and computer speed.

Now the database has been created, it can be used to create networks using MetaboNetworks. To start the software the main function has to be called from Matlab:

>> MetaboNetworks

This will ask the user to select a database if no cell structures exist in the workspace. Otherwise the user can select a cell structure with metabolite names of biomarkers. If a cell structure is found in the workspace, the SelectMetaboliteNamesGUI starts up. Here the user can select a list of names. When a list has been chosen, the software checks if there is a vector (double) in the workspace with the same dimensions as the selected cell structure. This association vector can contain information on the significance of the metabolites (e.g. correlation with the response variable), however this is optional. If no association vector is selected, the ‘Colour nodes based on association’ button is disabled in the MetaboNetworks GUI.


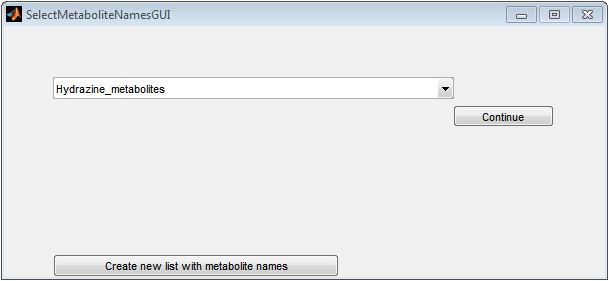


Alternatively, the user can create a new cell structure with metabolite names by clicking on ‘Create new list with metabolite names’ in SelectMetaboliteNamesGUI. This will ask the user to select a database to user for MetaboNetworks.


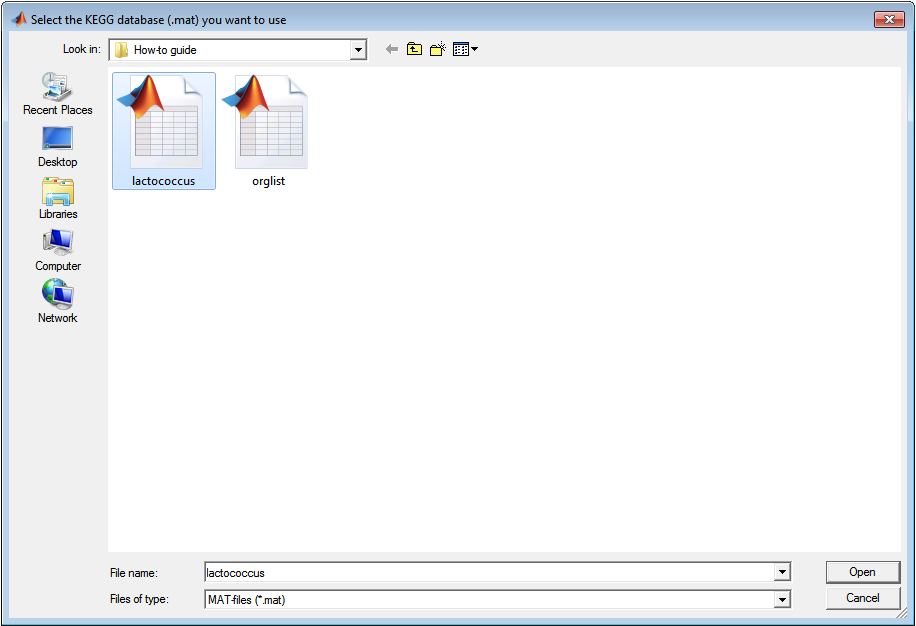


Here we select the database generated by MetaboGetworks and press open. If the user cancels, the software exits. If a valid database is selected, the SearchMetabolites dialog pops up. Here, the user can search for metabolites and add them to the list.


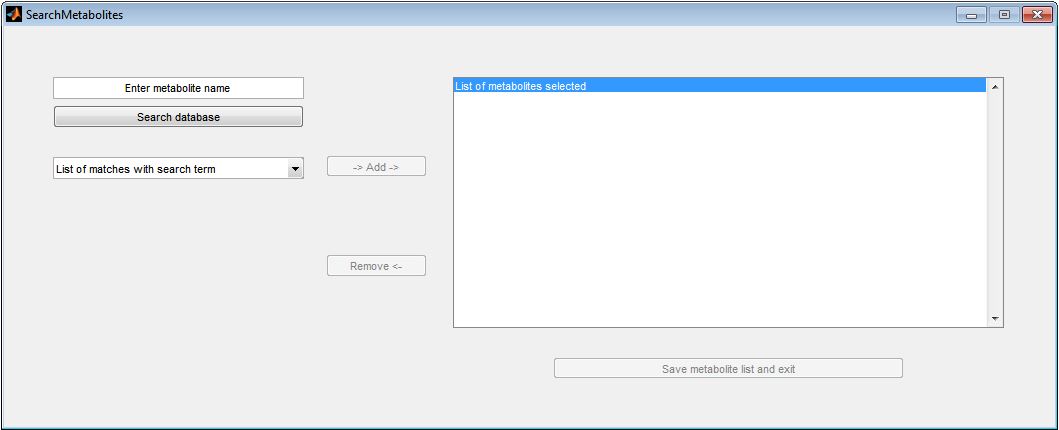


When multiple entries in KEGG match the search term, they are listed in a dropdown menu, after which the user can select the metabolite they wish to include.


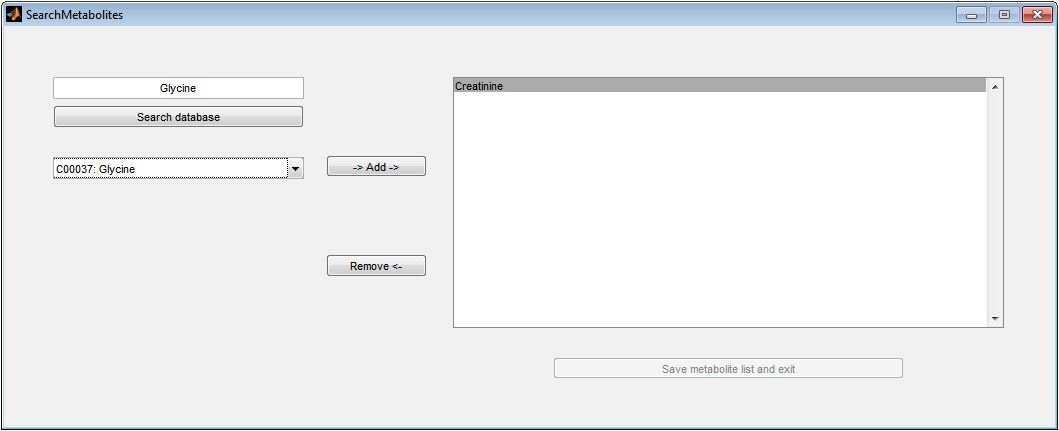


When more than 2 metabolites are selected, the user can save the list and exit.


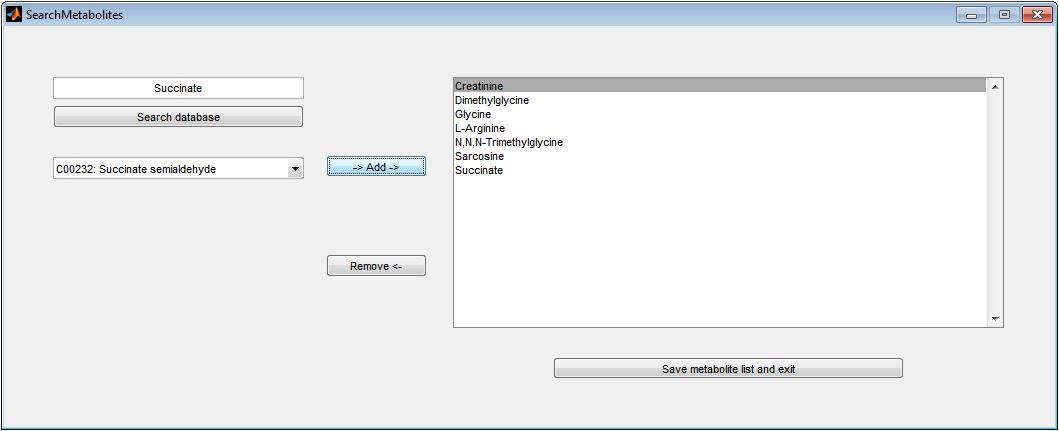


This will start up MetaboNetworksGUI with a default graph layout.


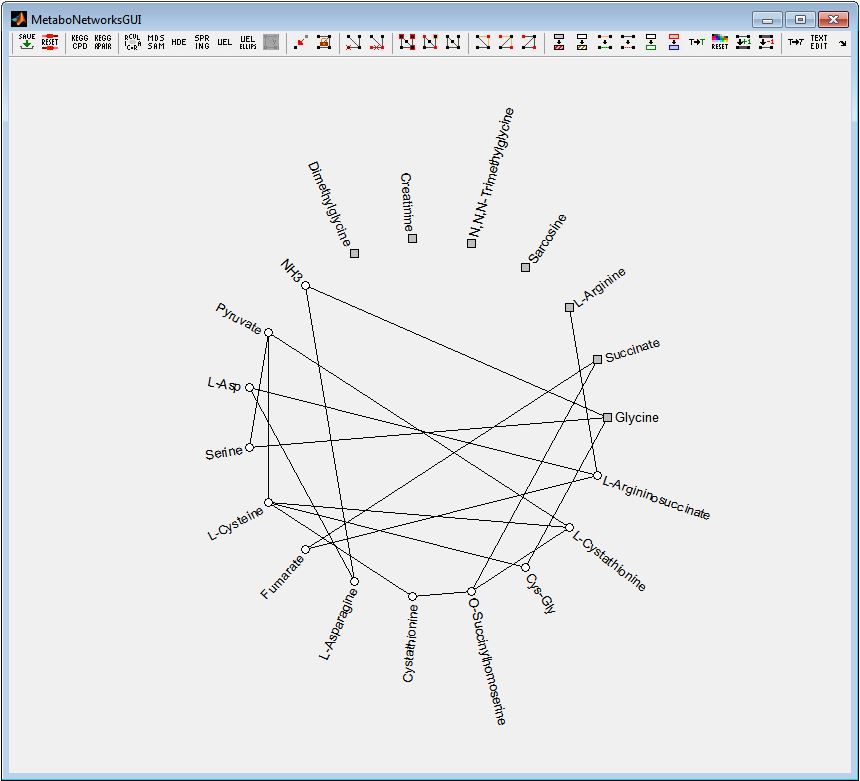


We have chosen to only include main reaction pairs in the database, however this can still result in probabilistic links that may not be wanted, e.g. the L-Asparagine – NH3 – Glycine path. The user can manually delete nodes from the network, in the example below NH3 was selected (red box colour of node) and when the delete button is pressed, MetaboNetworks immediately updates the network as shown in the next figure. The updated network shows some new additions to the shortest path network.


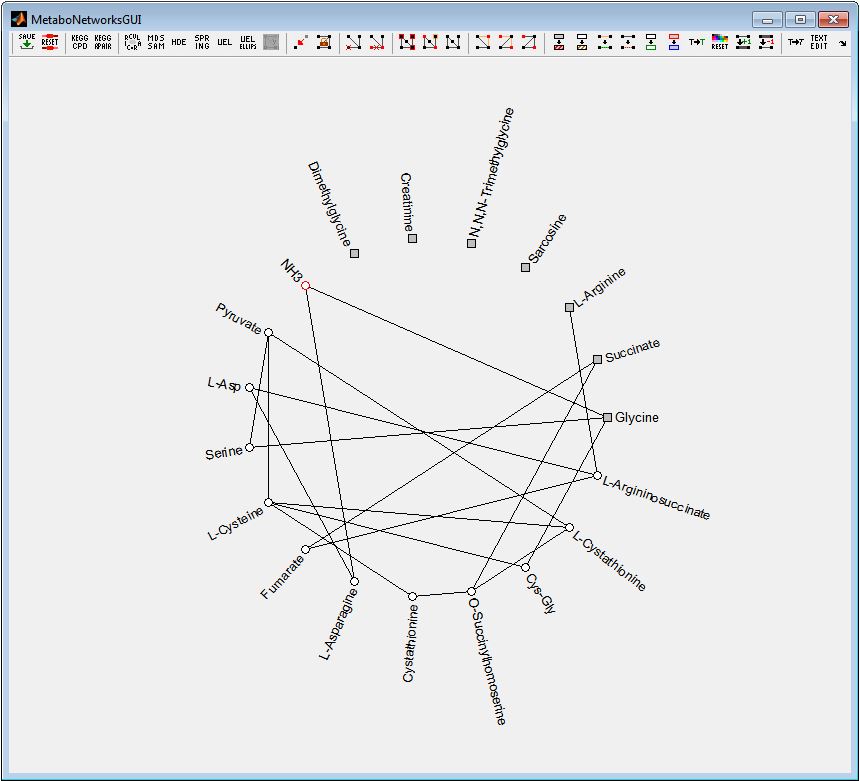

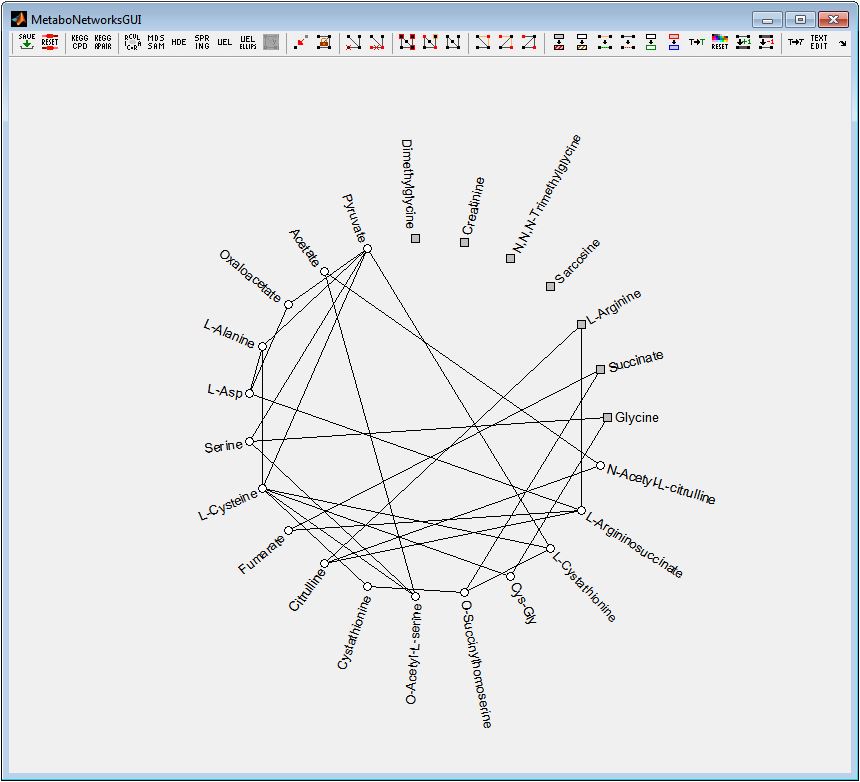


In order to investigate the shortest path between metabolites, we can select two metabolites (L-arginine and succinate were chosen here) and enable the shortest path button to highlight the shortest path.


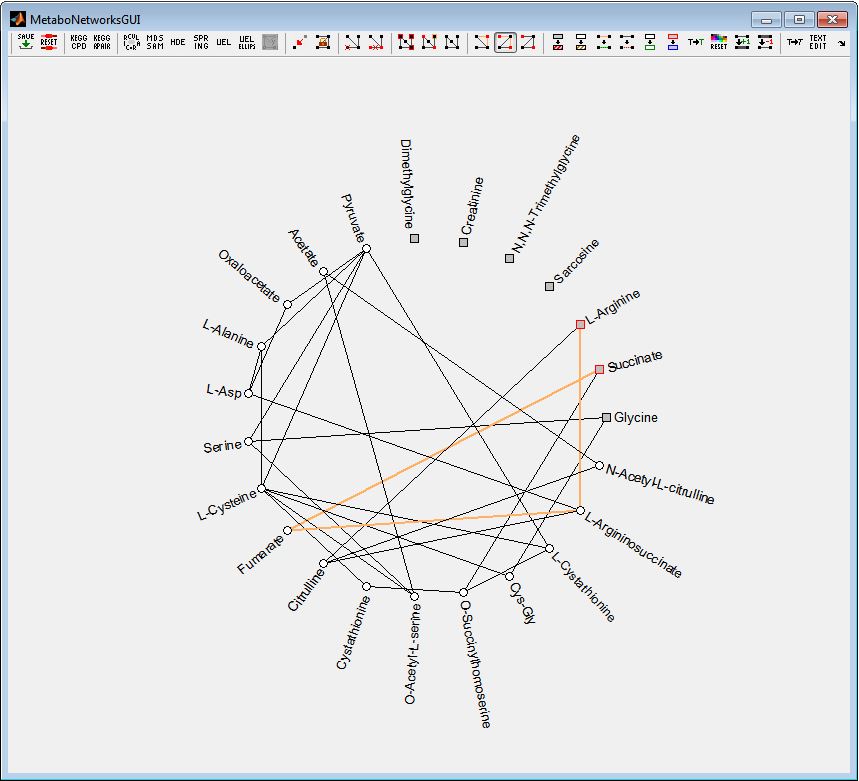


While the circular network is used as default, other graph layout algorithms can be used as well. Here we have chosen the spring-electrical embedding.


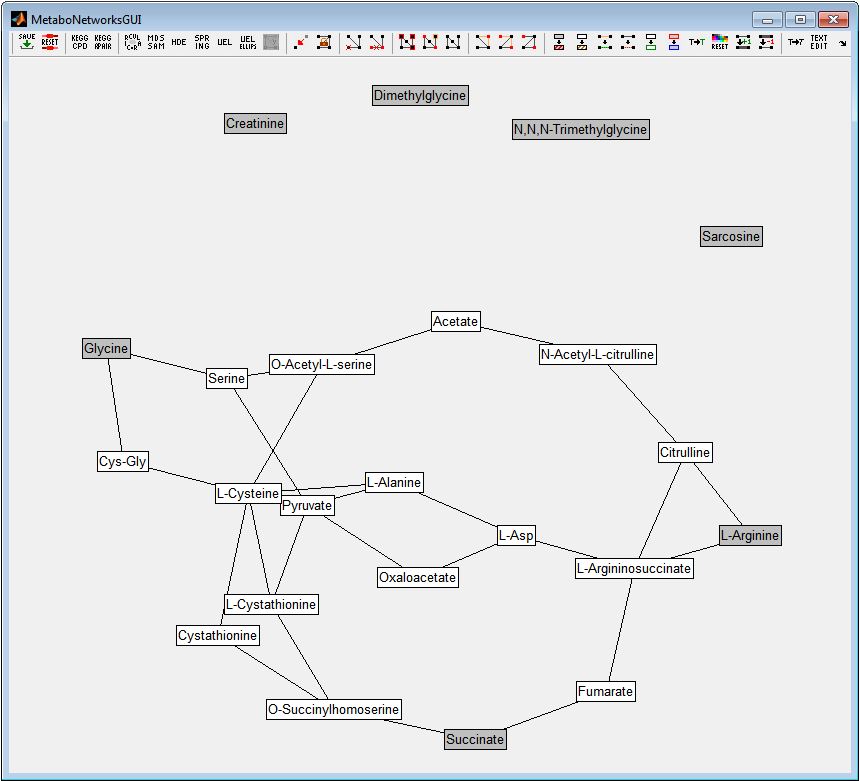


At any point the user can manually move nodes by enabling the move node button. We show here the result of moving the nodes to customize the figure so that there are no node labels overlapping.


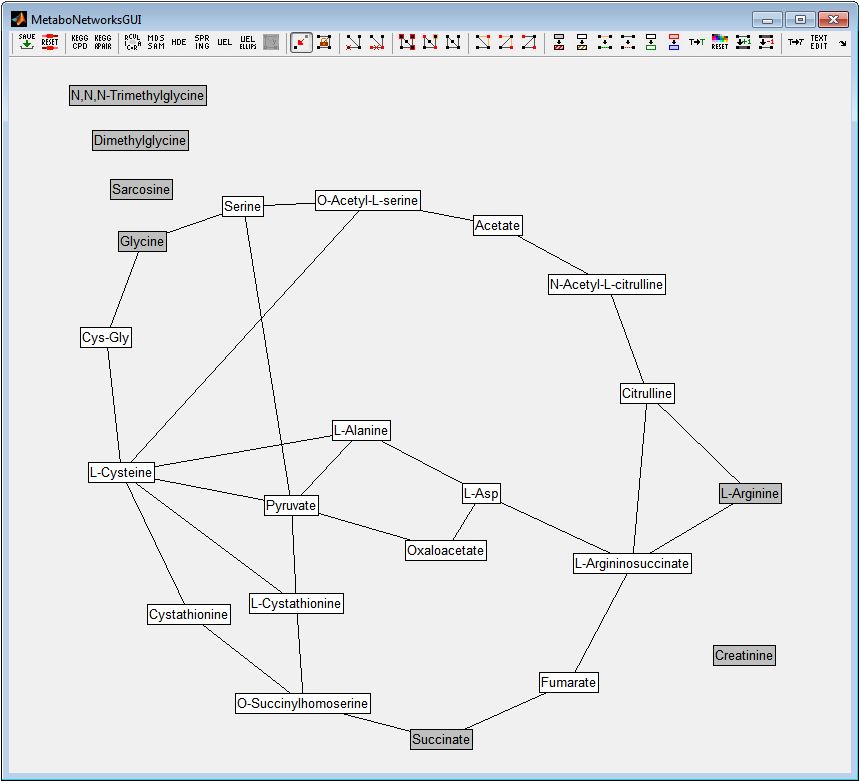


When the network becomes crowded (see figure 1), it may be beneficial to abbreviate metabolite names. MetaboNetworks uses the supplied biomarkers names and for all metabolites part of a shortest path the shortest entry in KEGG for the specific compound. When pressing ‘text edit’ and selecting a metabolite a dialog box pops up and the user can change the name. Alternatively, using the ‘repl. all’ button a specific search term can be replaced in all nodes containing it.


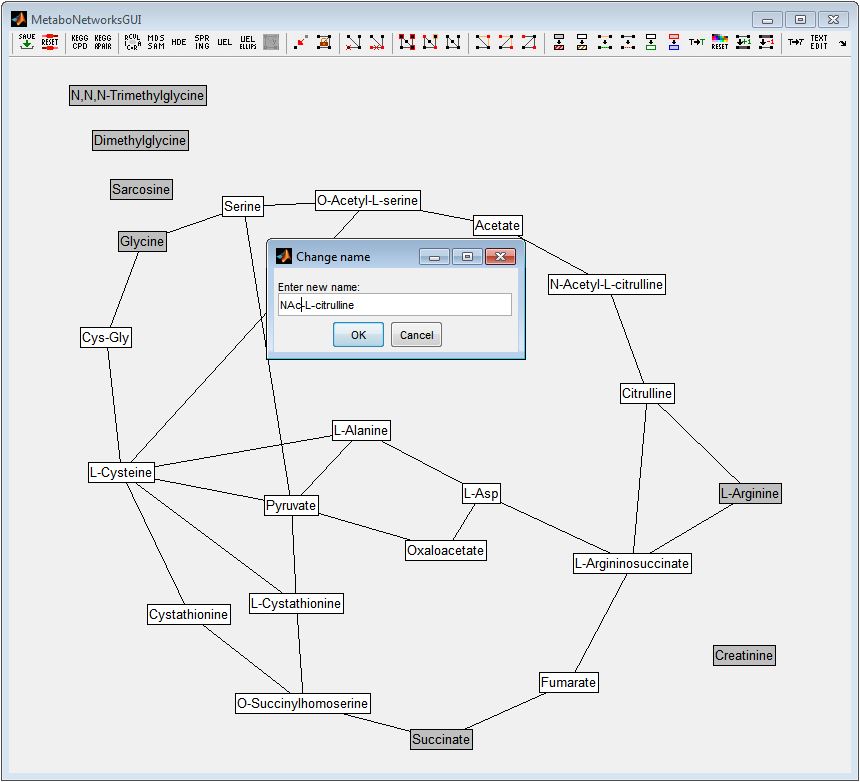


Alternatively, the user may want to change the default biomarker node colours. This will show a dialog box in which the user can select a custom colour.


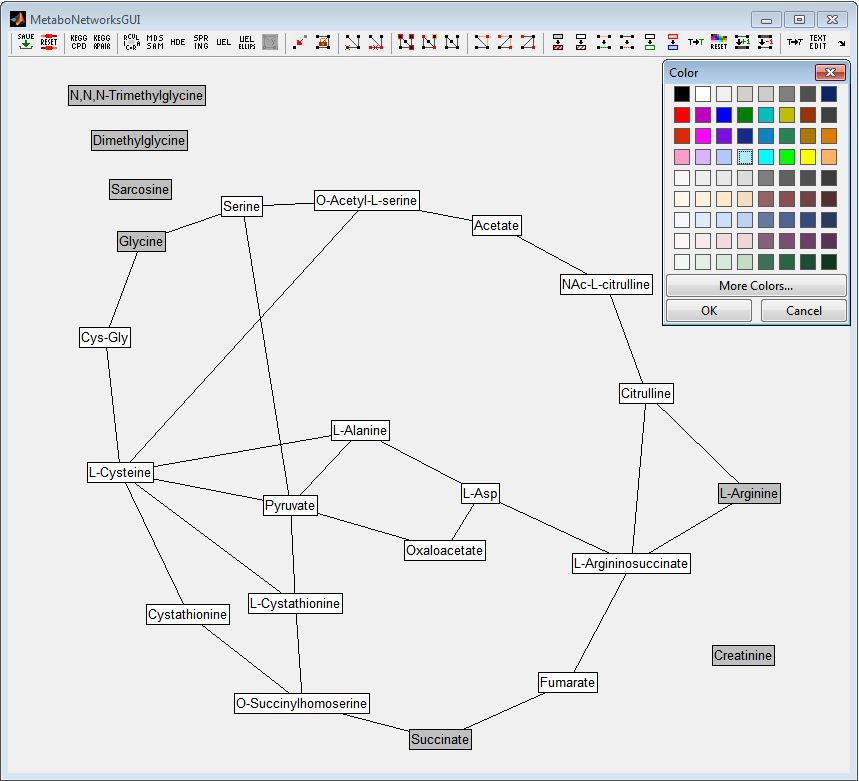


For clarity or publication the user may wish to change the font styles. A dialog box pops up when the T→*T* button is pressed.


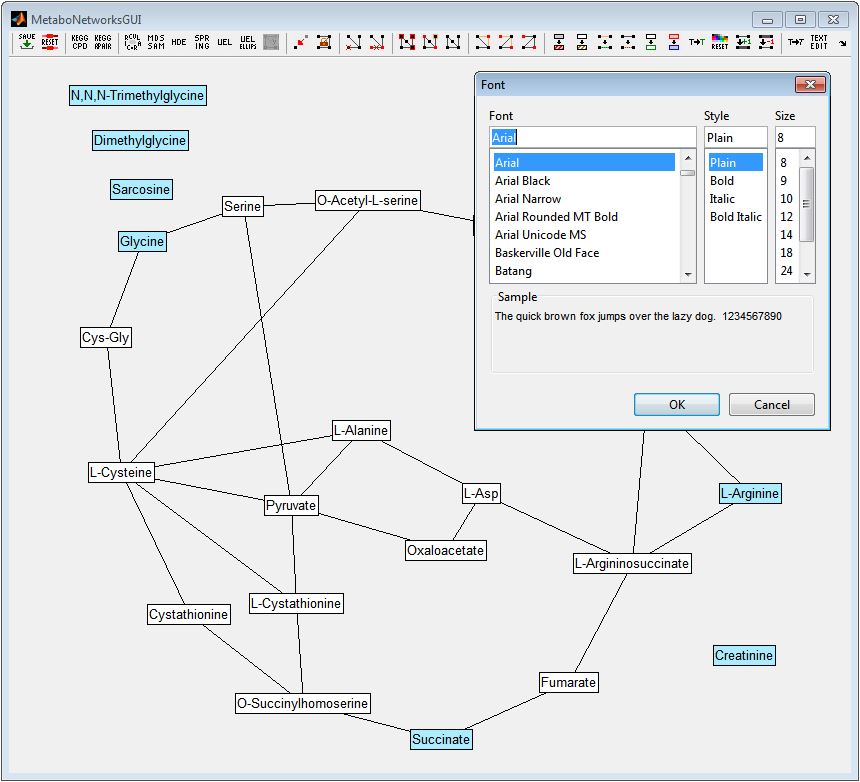


After changing font styles, nodes may overlap and need to be moved again for clarity.


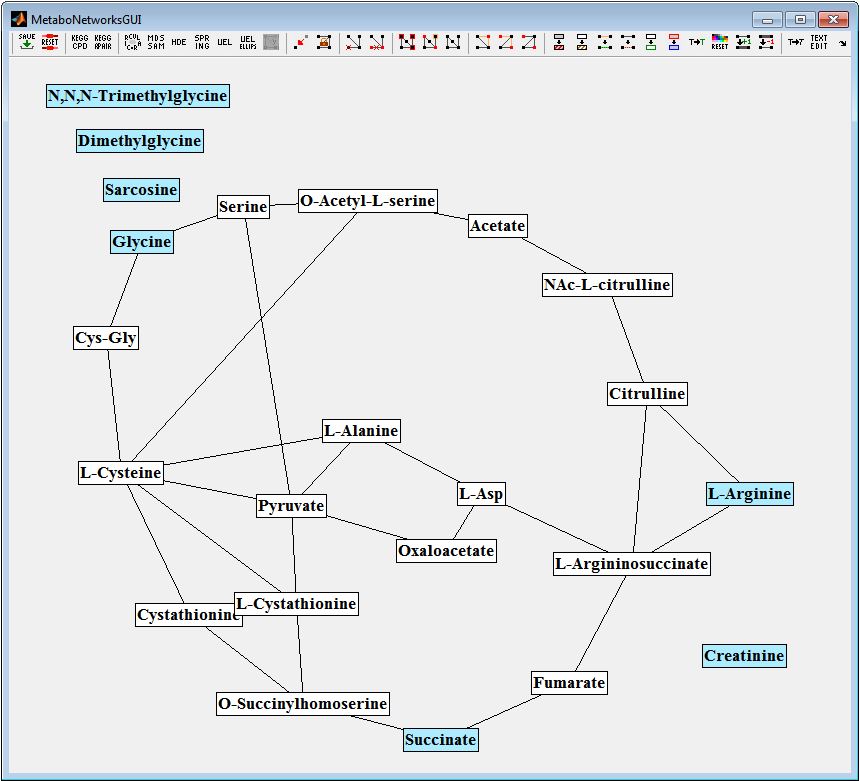


Also, the line widths can be changes with the +1 and -1 buttons.


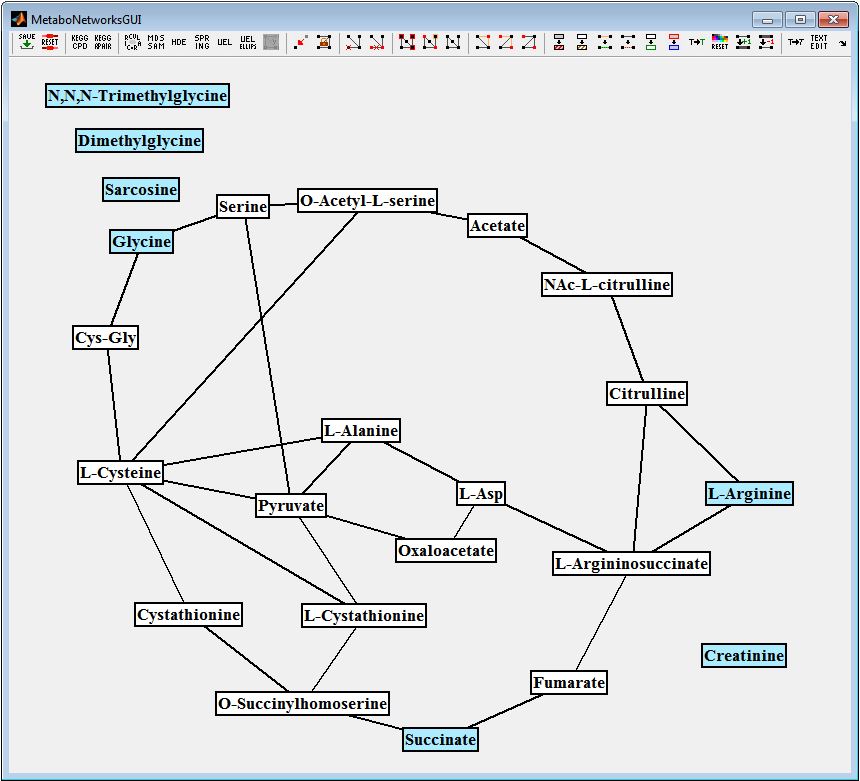


When the user is satisfied with the network it can be exported as an image when the save button is pressed. A dialog pops up in which the user can chose the file format, file name and location.


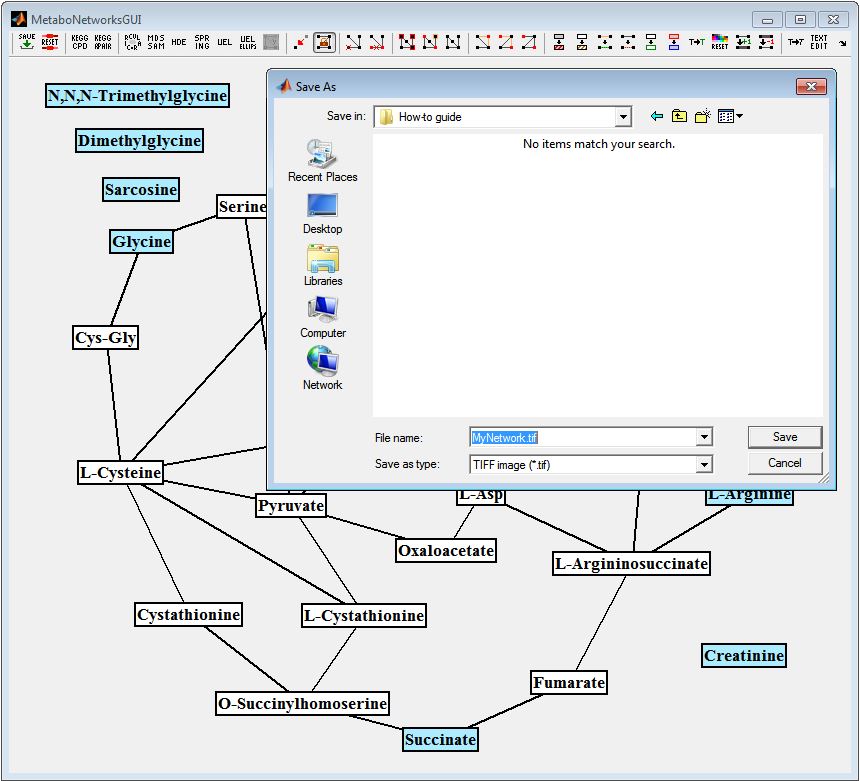


In order to explore the network, the user can select multiple nodes and look at the reaction pairs in KEGG. This opens a web browser and shows the reaction pairs if a link exists between selected metabolites.


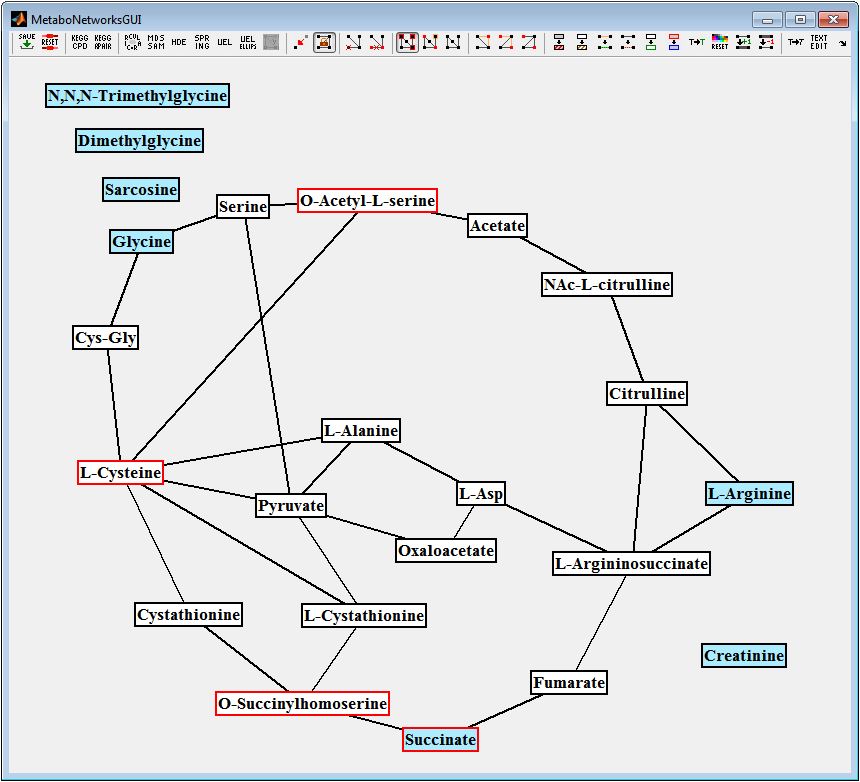


In this example, two links are found between selected metabolites and both reaction pairs are shown in the web browser.


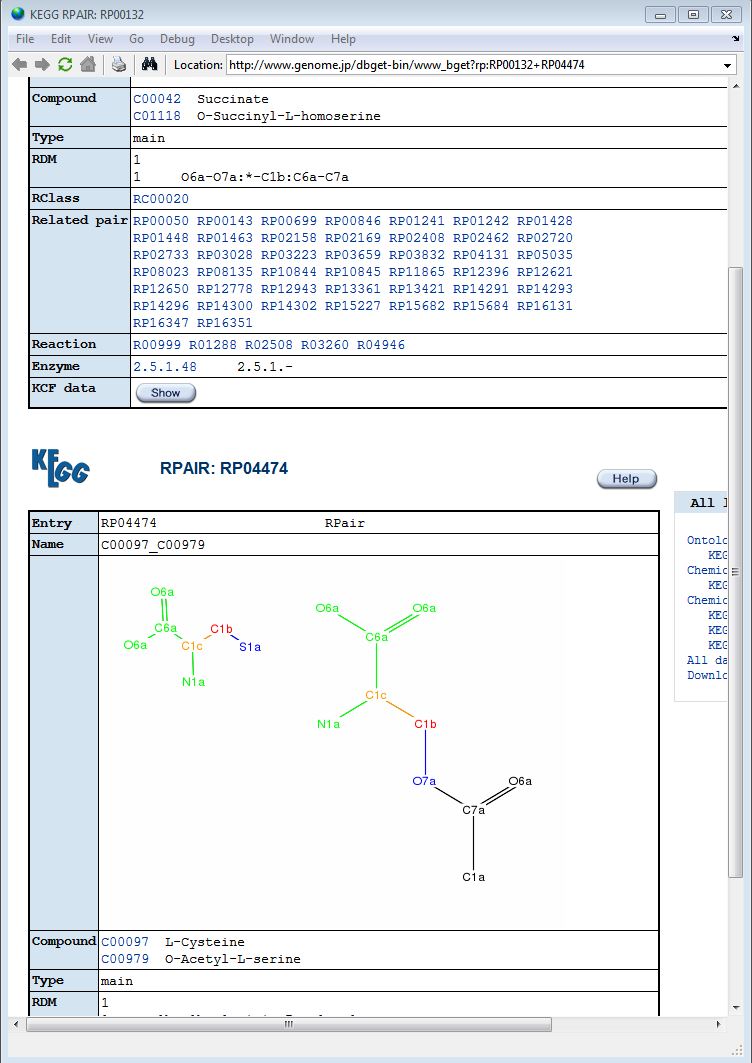


Alternatively the user can also select a single metabolite and open the corresponding compound page in KEGG.


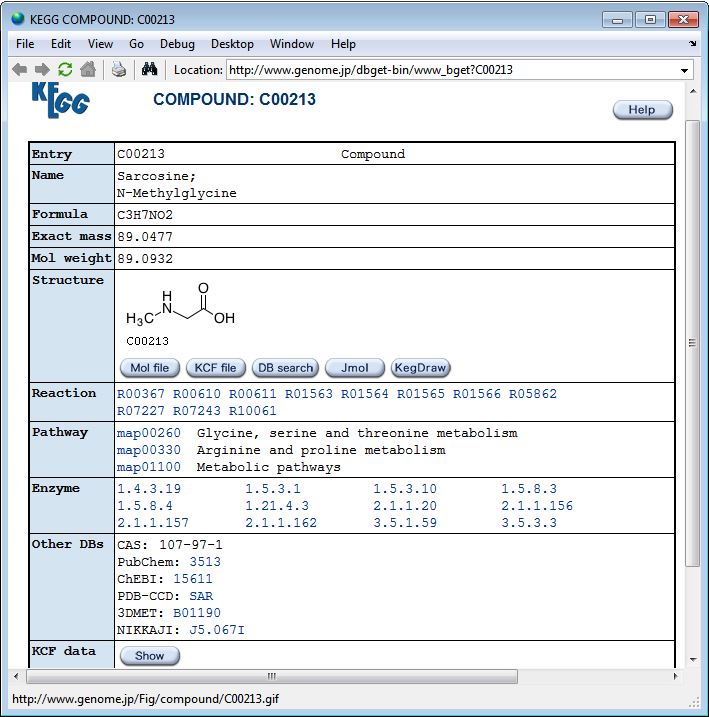


As an alternative to deleting a node from the network, the user can also delete edges between (selected) nodes. This will immediately calculate a new network layout and replace previous node positions (unlock).


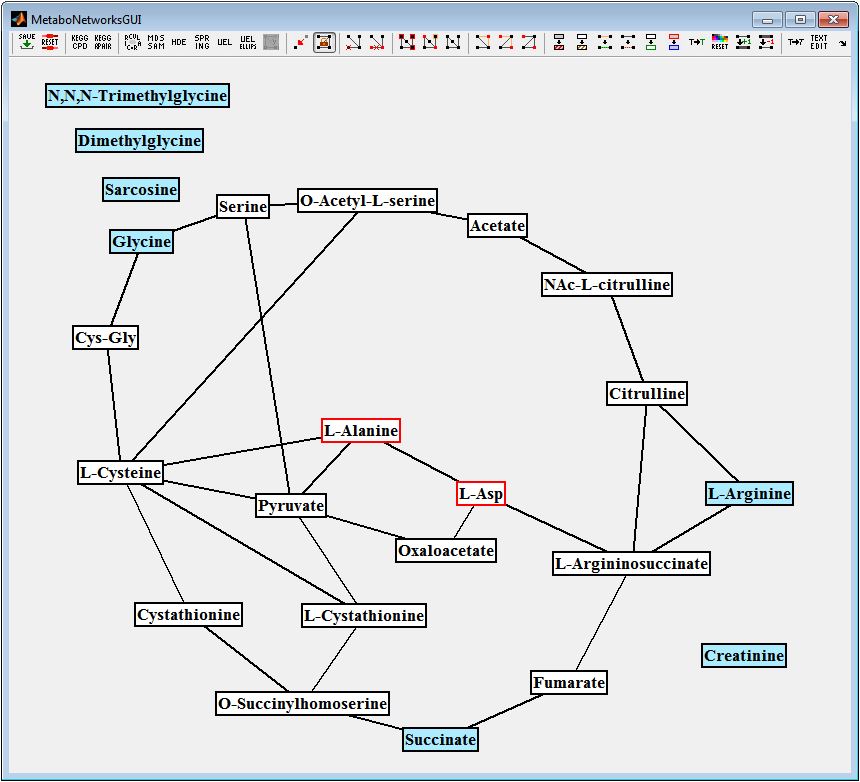


This has resulted in a new network where L-alanine is no longer present, but L-Asp still is as it is still part of a shortest path from L-arginine to glycine.


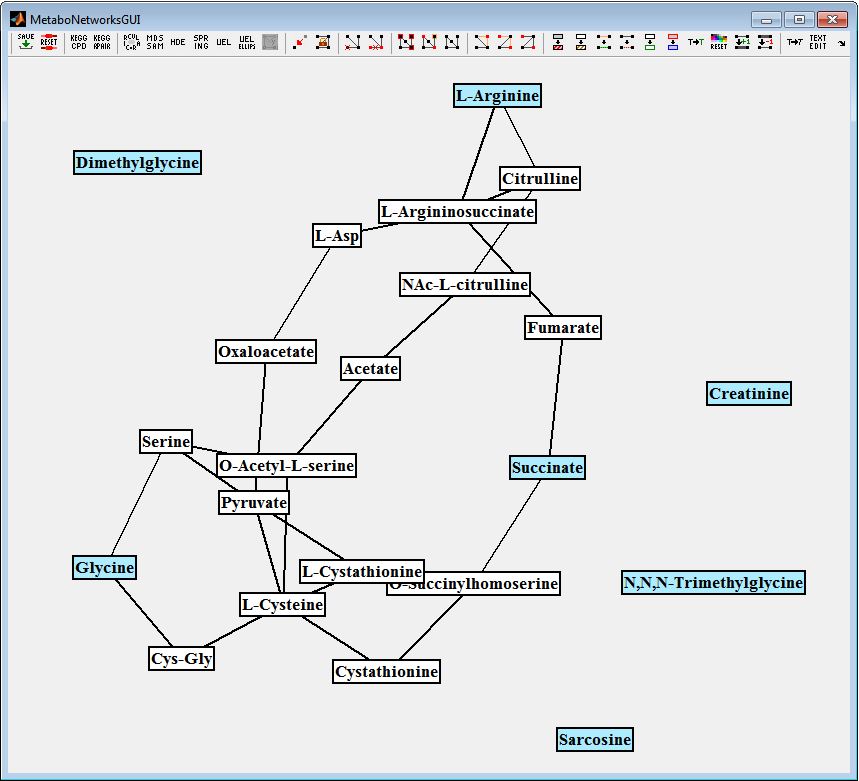

Supplement: Supplementary Data [file supp_btt612_MetaboNetworks_supplementary_information.doc]
